# Fertility Considerations in Adolescent Klinefelter Syndrome: Current Practice Patterns

**DOI:** 10.1210/clinem/dgz308

**Published:** 2019-12-26

**Authors:** Shalender Bhasin, Robert D Oates

**Affiliations:** 1 Harvard Medical School, Research Program in Men’s Health: Aging and Metabolism, Boston, Massachusetts; 2 Boston Claude D. Pepper Older Americans Independence Center, Brigham and Women’s Hospital, Boston, Massachusetts; 3 Boston University School of Medicine, Department of Urology, Boston Medical Center, Boston, Massachusetts

**Keywords:** Klinefelter syndrome, testicular sperm extraction, fertility preservation

In this issue of the Journal, Zganjar et al. (1) report the findings of a survey of the current practices of fertility care in adolescent males with Klinefelter syndrome (KS) among members of the Society for the Study of Male Reproduction, the Pediatric Endocrine Society, and the Endocrine Society (overall response rates of 4.7%, 5%, and 1%, respectively). Questions revolved around testosterone replacement, sperm banking, and testis sperm extraction (TESE) in adolescents with KS. Even though numerous publications have determined that testosterone levels in the adolescent KS male are within the low normal range (2), there continues to be debate about whether such males require testosterone replacement. Likewise, several thoughtful commentaries have discussed the pros and cons of TESE for “fertility preservation” during adolescence versus postponing procreative management until adulthood (eg, (3, 4)). In light of the relative paucity of high-quality evidence and a lack of consensus on the optimum timing of TESE in persons with KS (prepubertal, pubertal, adolescent, or adult), it is unsurprising that the survey conducted by Zganjar et al. (1) revealed wide variation in the approach to testosterone treatment and TESE in adolescents with KS.

KS, a group of chromosomal disorders characterized by the presence of 1 or more extra X chromosomes is typically associated with hyalinization of the seminiferous tubules, azoospermia, and infertility (2). The molecular basis of the KS phenotype is incompletely understood. Only some phenotypic features of KS can be explained by testosterone deficiency; other features have been attributed to the excess dosage effect due to overexpression of X chromosomal genes escaping inactivation and of additional X chromosome genes that exhibit variable cell type-specific expression. Skakkebaek et al. (5) identified unique epigenetic and genetic differences in the expression of both autosomal as well as X chromosome genes in men with KS compared with non-KS men. The genes that were differentially enriched in patients with KS relative to non-KS men and women included coding genes involved in immune regulation, Wnt signaling, and neuronal development, as well as noncoding genes involved in X chromosome inactivation (5). However, molecular mechanisms that contribute to accelerated apoptosis of germ cells with supernumerary X chromosome and the hyalinization of seminiferous tubules in men with KS remain unknown.

Even though all cells in the KS embryo carry a 47, XXY chromosomal complement, a small fraction of germ cells in the prepubertal testis are diploid (46, XY), likely due to a random loss of the extra X chromosome during 1 of many spermatogonial mitotic divisions (2, 6, 7). The vast majority of early germ cells in the testes of prepubertal boys with KS have a 47, XXY karyotype and suffer a meiotic block and undergo apoptosis shortly after puberty (2, 6, 7). Only spermatogonia with a normal 46, XY chromosomal complement survive and—under the influence of follicle-stimulating hormone and testosterone during and after puberty—complete the process of spermatogenesis and give rise to haploid sperm found in the rare islands of spermatogenesis discovered during TESE (2).

Men with KS have been historically considered sterile; however, the combination of TESE and intracytoplasmic sperm injection (ICSI), has enabled some men with KS to achieve fertility (8). A meta-analysis reported successful sperm retrieval in approximately 40% of men with KS; ~40% of those with successful sperm retrieval achieved a live childbirth (9). Thus, ~16% of men with KS, who undergo TESE and ICSI can likely achieve a live childbirth (9).

Damani et al. (10) demonstrated the feasibility of TESE in adolescent males with KS and wondered whether TESE should be considered in the adolescent KS as a means to optimize success rates and preserve biological paternity potential. However, since then, many studies have shown that sperm retrieval rates in the early adolescent are not as high as in the older adolescent which are, in turn, equivalent to those in the adult (~50%) (11). Therefore the literature does not support the mandatory need for TESE in the adolescent compared with simply waiting until the patient and his partner are desirous of conception. However, as can be seen by the survey results, this strategy is still seen as advantageous by some providers.

The clinical value the KS adolescent derives from testosterone treatment and the need for such therapy is based upon the notion that testosterone levels, which are typically normal or low normal in late puberty (12), decrease rapidly after puberty and that the KS male will eventually need testosterone replacement. However, the testosterone levels in the majority of adolescents with KS remain relatively stable into early adulthood (2, 12). Therefore, there is no need for reflex replacement in all men with KS (3). Since testosterone treatment would be expected to suppress whatever endogenous testosterone production and whatever minimal level of spermatogenesis might be present, harvesting and cryopreservation of sperm prior to starting testosterone replacement is indicated. However, there may be no need for TESE if there is no need for testosterone therapy. As seen by the survey results, however, a significant number of respondents (45%) favor testosterone treatment initiation even in the asymptomatic adolescent. Some of the endocrinologists in favor of early and universal testosterone treatment may be influenced by the persistently high luteinizing hormone and follicle-stimulating hormone as an indication of subclinical testosterone deficiency. However, the adverse health outcomes that might be associated with subclinical hypogonadism remain unclear; this may be a reason why neonatal screening for KS has not become commonplace.

Testicular sperm extraction is not without some downside; it is a surgical procedure that requires anesthesia, imposes some financial and emotional burden, and maybe associated with a decrease in circulating testosterone levels that may persist for 12 or more months after the procedure. Furthermore, it is often difficult for an adolescent boy to make an informed decision about future fertility which may appear abstract at that age. The adult with KS and his partner may be in a substantially better position to take into account the intensity of their desire to have a child, the success rates of TESE and ICSI, and the financial and emotional burdens of a surgical procedure on the patient and that of the treatment cycle to harvest oocyte from his partner.

Thus, there is no compelling reason that TESE has to be performed during adolescence (3, 4) and no definitive evidence that postponing the procedure to early adulthood worsens outcomes. However, it should be recognized that the choices made by men with KS and their partners and families are influenced by many factors—including important nonmedical considerations—such as the natural longing of men and women to have children, complex social and cultural pressures, and the financial and emotional burden associated by these procedures. Furthermore, individual patients with KS and their partners may weigh the trade-offs between the probability of successful treatment outcome (~16%) and the risks and costs associated with TESE and ICSI differently depending upon their unique life situations. Ultimately, such complex life decisions require individualized and shared decision making, especially when the available evidence is so limited and of variable quality.

## Abbreviations

ICSI: intracytoplasmic sperm injection
KS: Klinefelter syndrome
TESE: testis sperm extraction

## Additional Information

***Disclosure Summary:*** Dr Bhasin reports receiving grant support for investigator-initiated studies, unrelated to this article, from NIA, NINR, NICHD, and FNIH; from AbbVie, Alivegen, Transition Therapeutics, and MIB; consulting fees from OPKO; and he holds equity interest in FPT, LLC. These conflicts are managed and overseen by the Office of Industry Interaction of the Brigham and Women’s Hospital and Partners Healthcare System. Dr Oates has no disclosures.
